# What to expect from your remote eye-tracker when participants are unrestrained

**DOI:** 10.3758/s13428-017-0863-0

**Published:** 2017-02-15

**Authors:** Diederick C. Niehorster, Tim H. W. Cornelissen, Kenneth Holmqvist, Ignace T. C. Hooge, Roy S. Hessels

**Affiliations:** 1 The Humanities Laboratory, Lund University, Lund, Sweden; 2 Department of Psychology, Lund University, Lund, Sweden; 3 Institute for Psychology, University of Muenster, Muenster, Germany; 4 Scene Grammar Laboratory, Department of Cognitive Psychology, Goethe University Frankfurt, Frankfurt, Germany; 5 UPSET, North-West University (Vaal Triangle Campus), Vanderbijlpark, South Africa; 6 Experimental Psychology, Helmholtz Institute, Utrecht University, Utrecht, The Netherlands; 7 Developmental Psychology, Utrecht University, Utrecht, The Netherlands

**Keywords:** Eye-tracking, Head movement, Head orientation, Developmental studies, Data quality, Clinical groups

## Abstract

The marketing materials of remote eye-trackers suggest that data quality is invariant to the position and orientation of the participant as long as the eyes of the participant are within the eye-tracker’s headbox, the area where tracking is possible. As such, remote eye-trackers are marketed as allowing the reliable recording of gaze from participant groups that cannot be restrained, such as infants, schoolchildren and patients with muscular or brain disorders. Practical experience and previous research, however, tells us that eye-tracking data quality, e.g. the accuracy of the recorded gaze position and the amount of data loss, deteriorates (compared to well-trained participants in chinrests) when the participant is unrestrained and assumes a non-optimal pose in front of the eye-tracker. How then can researchers working with unrestrained participants choose an eye-tracker? Here we investigated the performance of five popular remote eye-trackers from EyeTribe, SMI, SR Research, and Tobii in a series of tasks where participants took on non-optimal poses. We report that the tested systems varied in the amount of data loss and systematic offsets observed during our tasks. The EyeLink and EyeTribe in particular had large problems. Furthermore, the Tobii eye-trackers reported data for two eyes when only one eye was visible to the eye-tracker. This study provides practical insight into how popular remote eye-trackers perform when recording from unrestrained participants. It furthermore provides a testing method for evaluating whether a tracker is suitable for studying a certain target population, and that manufacturers can use during the development of new eye-trackers.

Remote eye-trackers are becoming increasingly popular because they are easy to set up and enable the measurement of where a person looks while allowing free head movement. A researcher’s decision of which eye-tracker to use is often guided by the information provided by manufacturers about their machine’s spatial *accuracy* (the average offset between the point on screen that the participant looks at and the point reported by the eye-tracker), its *precision* (the sample to sample difference in what the eye-tracker reports when the gaze is fixed on a point on the screen), and the *headbox* (the dimensions of the volume in front of the eye-tracker in which tracking is possible). The marketing material of eye-tracker manufacturers suggests that these values are representative of a participant anywhere inside the eye-tracker’s headbox, or makes no mention of possible negative consequences of non-optimal head positions and orientations (SMI, 2015; SR Research, 2016; Tobii, 2016). However, our practical experience indicates that the values provided by manufacturers are only representative of participants who follow instructions carefully and sit still in an optimal position and orientation. When participants assume non-optimal poses (Hessels et al., 2015b), or when recording from difficult participant groups such as infants (Hessels et al., 2015a), accuracy and precision suffer and *data loss*, the period of time during which the eye-tracker is unable to report a gaze position, can be substantial, even if the participant’s eyes remain in the headbox. Reduced data quality poses important problems for data analysis (Hessels, Niehorster, Kemner, & Hooge, 2016).

Nonetheless, manufacturers present data quality measures in their marketing materials that were acquired under optimal conditions. While most manufacturers such as SMI and SR Research provide little information about how they arrive at their advertised values, Tobii’s material for the TX300 states the following: “*The Tobii Pro TX300 collects gaze data at 300 Hz while allowing for large head movements. This unique capability, together with extremely-high accuracy and precision, as well as vast tracking robustness, extends the possibilities for unobtrusive research into human behavior and oculomotor functions. In both behavior and eye-movement research involving children or primates, subjects can be positioned comfortably, without an unnatural chinrest.*” (Tobii, 2016). However, the fine print in the technical specifications of this tracker clarifies that the accuracy values presented in the marketing material are only achieved “*under ideal conditions […] measured in the centre of the head movement box with the subject fixed in a chinrest. Data is collected immediately after calibration, in a controlled laboratory environment with constant illumination, with 9 stimuli points at gaze angles of ≤18°. Measurements are done on […] subjects without lenses, glasses or droopy eyelids*” (Tobii, 2010, p. 17), whereas for the marketed precision values the human subject was even replaced with an artificial eye (Tobii, 2010, p. 17). This begs the question of whether the specifications provided by manufacturers are still relevant when the subject moves, or even when they sit still but are not restrained by a chinrest.

In this paper, we therefore investigate how a selection of remote eye-trackers performs when data are recorded in non-optimal conditions. There are many participant groups that cannot be restricted on a chinrest or cannot be instructed to sit still, such as infants, attention-deficit hyperactivity disorder (ADHD) patients, or clinical groups such as Alzheimer and Parkinson patients, and people with muscular disorders. Using a remote eye-tracker can make it possible to record the gaze behavior of these groups, but it is important to understand the consequences of their failure to sit still (whether by inability or by not following instructions) for data quality. Personal observations indicate that even healthy university students have trouble remaining in an optimal pose when not restrained—in fact, the need to sit still at least partly defeats the purpose of a remote eye-tracker. Knowledge of how the eye-tracker copes under these conditions is invaluable when selecting the eye-tracker you will use for your study, particularly when the manufacturer’s specifications may not be informative enough.

Most studies where eye-tracker performance is assessed do so in optimal conditions when participants sit still (e.g., Nyström et al., 2013). In contrast, Hessels et al. (2015b) have provided the first comparison of remote eye-tracking performance in non-optimal conditions where participants moved or held their head in various orientations. Their results revealed that there are marked qualitative differences in how remote eye-trackers cope with these non-optimal conditions, both between manufacturers and between the different systems of individual manufacturers. Since Hessels et al.’s (2015b) work, significant development has occurred among remote eye-trackers with manufacturers releasing several new trackers and using the results from Hessels et al.’s (2015b) study to improve their eye-trackers. Here, we thus extend Hessels et al. (2015b) by evaluating five remote eye-trackers that were omitted from their study, have been brought to market since their study, or that the manufacturers claim have been improved to provide more robust data from participants in non-optimal poses. We furthermore provide a wider range of tests of remote eye-tracker performance in non-optimal conditions. Specifically, we evaluate eye-tracker performance during head orientations rotated around all three axes instead of the two axes examined by Hessels et al. (2015b), as personal experience with infants informs us that head rotation around all three axes can detrimentally affect data quality. In addition, we assess remote eye-tracker performance in more recovery scenarios in which tracking of one or two eyes is lost, and we adopt a more detailed analysis to provide insight into how the eye-trackers keep track of the eyes during data loss and recovery. We wish to emphasize that these testing conditions are designed to uncover problems in how eye-trackers cope with the non-optimal conditions that occur in real research settings. In this paper, we will focus on substantial problems, such as for instance large offsets in the measured gaze signal, instead of evaluating eye-trackers’ spatial accuracy and precision in detail.

In this study, we compare the performance of five different remote eye-trackers. These are (1) the Tobii Pro TX300 as Hessels et al. (2015b) found that it showed the least data loss and smallest offsets during non-optimal head orientations among the trackers in their test; (2) the EyeLink 1000Plus in remote configuration as it is increasingly used as a remote eye-tracker in, for instance, infant labs (e.g., Óturai, Kolling, & Knopf, 2013; Van Renswoude, Johnson, Raijmakers, & Visser, 2016) and it is advertised as providing the highest spatial accuracy and precision among video-based trackers; (3) the SMI REDn Scientific as according to the manufacturer it is their best eye-tracker for non-optimal conditions as it has the largest headbox among its trackers, and it is a new model in which problems reported by Hessels et al. (2015b) for older SMI products have been fixed according to SMI; (4) the Tobii T60XL as according to the manufacturer it is has the largest headbox among its trackers; and (5) the EyeTribe, a popular low budget eye-tracker. This study provides the reader with practical insight into how a selection of popular remote eye-trackers perform when used to record from unrestrained participants. We investigate the robustness with which eye-trackers report data during tasks that simulate various non-optimal participant poses that can occur during experiments with unrestrained participants. Whereas the manufacturers’ specification sheets say little about eye-tracker robustness when recording from unrestrained participants in non-optimal poses, the performance of the eye-trackers in our tasks enable the reader to make an informed decision regarding which remote eye-tracker is most suitable for their experiments. Furthermore, this work provides a testing method that can be used to acquire meaningful and easily understood performance measures on your remote eye-tracker in real-life conditions, allowing the reader to further evaluate whether a tracker is suitable for their study with their target population.

## Method

### Participants

Seven volunteers (three naïve to the specific goals of the study and four authors; five males, two females) between the ages of 25 and 51 years participated in the experiment at Lund University. All participants had normal or corrected to normal vision and provided informed consent. All participants had previous experience with participating in eye-tracking research. Information was recorded regarding the participants’ eyelash direction, eye color, eyelid coverage, and whether they had glasses or contacts, following a coding scheme similar to that of Nyström et al. (2013) and  Hessels et al. (2015a). Information about the participants is presented in Table 1.

**Table 1:**
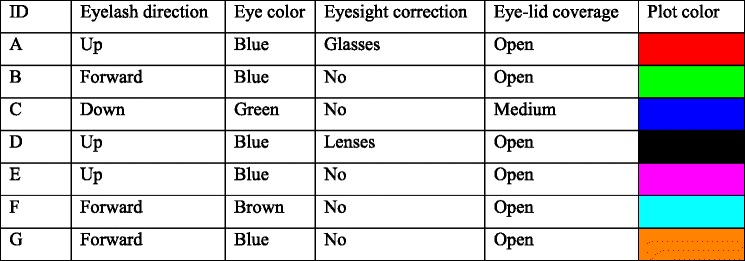
Information about the participants, along with the color in which their data are plotted

### Apparatus

Five different eye-trackers from SMI, SR Research, The EyeTribe, and Tobii were used, as listed in the introduction section. Their specifications as provided by the manufacturers are listed in Table 2. Each of these eye-trackers was set up according to the manufacturer’s recommendations. The SMI REDn was attached underneath the screen of the laptop that comes with it. All operators were experienced with the eye-tracker they operated. DN (first author) operated the SMI and EyeLink, together with TC (second author). TC operated the EyeTribe, and RH (last author) operated both Tobiis. Following the manufacturer’s recommendations in the manual, the EyeLink was equipped with the 16-mm lens for this test.

**Table 2:** Specification of each eye-tracker as provided by the manufacturers

	SR Research EyeLink 1000Plus	The EyeTribe	SMI REDn	Tobii T60XL	Tobii Pro TX300
Sampling frequency (Hz)	500	30	60	60	300
Accuracy (°)	0.5	0.5−1	0.4	0.5	0.4
Precision (° RMS)	<0.05	0.1	0.05	0.35	0.15
Headbox width × height (cm)	40 × 40 (at 70 cm)	40 × 30 (at 65 cm)	50 × 30 (at 65 cm)	44 × 22 (at 70 cm)	37 × 17 (at 65 cm)
Headbox depth (cm)	50−70	45−75	40−100	50−80	50−80
Recommended tracking distance (cm)	55−60	Not specified	Not specified	65	65
Requirements for remote mode	Sticker on forehead	None	None	None	None

Stimulus presentation was done with MATLAB using the Psychophysics Toolbox (Brainard, 1997; Pelli, 1997). For the SMI and Tobii machines, their SDKs were used for data recording. For the EyeLink, the EyeLink toolbox for MATLAB (Cornelissen, Peters, & Palmer, 2002) was used and for the EyeTribe, software that was part of the PyGaze toolbox (Dalmaijer et al., 2014) was used. Offline analysis of the data files was conducted in MATLAB.

### Procedure

Before the start of each task, participants were positioned in front of the eye-tracker at the manufacturer’s recommended distance (see Table 2), using the distance values reported by the manufacturer’s software. Then, a calibration was run using the default calibration setup of the manufacturer. No fixed criteria for judging calibration quality across the five systems were used. Instead, the operators used their experience to decide what constitutes good quality for a specific setup, and took the best calibration they were able to achieve. Specifically, for SMI, a five-point calibration was used, followed by a four-point validation routine. Calibration was judged successful if the offset reported by the SMI SDK was less than 1°. For the EyeLink, a nine-point calibration routine was used followed by a nine-point validation routine. We accepted the lowest offsets we could get after multiple calibration attempts. For the EyeTribe, a nine-point calibration routine was used followed by a nine-point validation routine. We accepted calibrations where the EyeTribe software reported “good” for all validation points. For the two Tobii trackers, a five-point calibration was run, followed by inspection of the calibration results from the Tobii SDK, performed by a researcher (RH, last author) with multiple years of experience using Tobii eye-trackers.

After positioning and calibration, participants were given one of six tasks, in counterbalanced order. These six tasks were designed to investigate the three main research questions of the present article. First, we examined how the eye-trackers recovered after having lost track of the eyes. Second, we investigated whether eye-trackers reported data when one eye disappears, and how eye-trackers recovered when an eye reappears that is different from the eye that was last lost. Third, we examined how the eye-trackers performed when participants adopted non-optimal head orientations.

#### Recovery tasks

**Fig. 1:**
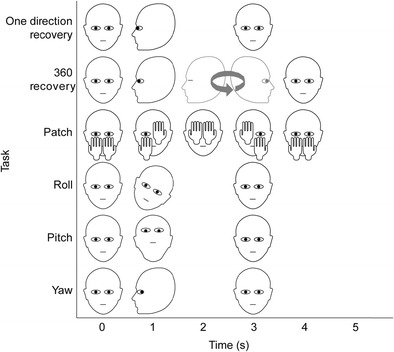
Schematic representation of the head orientations and hand positions over time during the six tasks. The black-outlined heads represent the poses participants were instructed to adopt at that time during the trial. Movements were timed by means of auditory beeps at 1 and 3 s for the one direction recovery, roll, pitch, and yaw tasks; beeps at 1 and 4 s for the 360 recovery task; and beeps at 1, 2, 3, and 4 s for the patch task. No beeps where played at the start of the trial. During the 360 recovery task, the head was turned with the eyes closed at 2–3 s, but this movement was not timed by auditory beeps

Two different recovery tasks were performed by the participants (see Fig. 1). For the first, dubbed the *one direction recovery* task, participants were asked to fixate a dot at the center of the screen at the start of each 5-s trial. After 1 s, a low-pitched beep signaled that participants should rotate their head and look away from the screen toward one of two poles that were placed directly to the left or the right of the participant, at 90° away from looking straight forward. Two seconds later, a high-pitched beep sounded, which instructed participants to rotate their head back and fixate the dot at the center of the screen. With this task, we investigated what happens when an eye-tracker lost track of the eyes and what happens when it restarts reporting gaze data. Note that in this investigation we focused on what the reported gaze signal looks like when an eye-tracker loses or regains track, not on the speed with which it recovered.

The second task, the *360 recovery* task, was similar to the regular recovery task, except that the participants returned to the screen from the other side than where their eyes left the screen, mimicking a 360° turn. Specifically, participants started by looking at the fixation point at the center of the screen, until at 1 s a low-pitched beep signaled them to look away from the screen and toward a pole at one of their sides. After this, they were asked to close their eyes and rotate their head to face the pole at their other side. Three seconds after the low-pitched beep, a high-pitched beep sounded, which instructed participants to rotate their head back and fixate the dot at the center of the screen. This task was designed because we know from previous work (Hessels et al., 2015b) that eye-trackers vary in how they recover when gaze returns to the screen, and we wanted to provide a more difficult test to further test their recovery abilities.

#### Eye-patch task

In the *eye-patch* task, participants were instructed to first occlude their eyes one at a time by covering them with their hands, and then reveal them again one at a time (see Fig. 1). Specifically, participants started the trial with their hand placed with their fingertips below their cheekbones. During the 5-s trial, several beeps sounded, instructing the participants to move their hands. After 1 s, the first beep sounded signaling participants to raise their first hand to occlude the left eye. After another second, a second beep sounded indicating that the right eye should be occluded with the other hand. After a further second, a third beep signaled to reveal the left eye by lowering the first hand. Finally, after another second a last beep sounded to indicate that the second hand should be lowered to reveal the right eye. The task was also performed in a sequence in which the right eye was covered and revealed first. A notable feature of this task is that, with this order of hand movements, the eye that is first revealed is different from the eye that last disappeared from the eye-tracker’s view. We designed this task to look at whether the eye-trackers are able to continue recording when one eye disappears, and can resume recording when an eye reappears that is different from the eye that was last lost.

#### Head orientation tasks

Participants completed three different head orientation tasks, in which they rotated their head as far as possible around one of the three rotation axes while maintaining continuous fixation on a dot at the center of the screen (see Fig. 1). In the *yaw* orientation task, participants were instructed to turn their head to the left or to the right. In the *pitch* task, participants were instructed to tilt their head upward or downward. In the *roll* task, participants tilted their head leftward or rightward. Trials lasted 5 s for all three tasks. Participants started in a normal viewing pose with their head upright and oriented straight ahead. After 1 s, a low-pitched beep indicated that they should rotate their head as far as possible along one of the three axes while maintaining fixation on the central dot. After a further 2 s, a high-pitched beep indicated that they should rotate back to the initial viewing pose. The main research question motivating these orientation tasks is to examine how the eye-tracker copes with participants who assume non-optimal head orientations. Specifically, we will investigate whether the eye-tracker is able to report a gaze position when the participant is in a rotated pose, and if so, whether the accuracy of the gaze signal suffers as shown by offsets in the reported gaze position data when the head is rotated compared to the normal upright and straight-forward head position. Rotations around each of these axes occur during real experiments with remote eye-trackers. For example, participants make yaw rotations when looking away from the screen, pitch rotations when sliding down in the seat and roll rotations when resting one of their cheeks on one of their hands.

For all tasks, the movements were practiced in mock trials before the recording of experimental trials was started. For all tasks, participants completed ten trials in each direction. Participants started with ten trials where they rotated their head leftward, tilted their head upward, or first put up their left hand. After these ten trials, three short beeps were sounded to tell participants to switch to rotating their head rightward, tilting their head downward or first putting up their right hand for another ten trials. Note that to minimize direction errors and variability in movement execution, the order of movement directions was not counterbalanced between tasks or between participants. No instructions were given to participants with regard to blinking their eyes. One participant was recorded with a 240 frames/s video camera as she performed the six tasks in front of the first and the last eye-tracker to check for differences in timing or head orientation. With the exception of the first trial of each task, it was found that the latency and duration of the initial movement of each task were identical (same number of frames in the 240-Hz video recording) in the first and last eye-tracker, for each task. The participant appeared to have found a consistent rhythm that was driven by the beeps. Although there certainly is between-subjects variation in the latency and duration of movement execution, the execution of the movements was most likely done the same by each participant for each eye-tracker. This means that any differences we find in the data between eye-trackers are likely for a large part due to the eye-tracker, and not due to participant variability.

### Data analysis

In our plots, we display gaze position as an offset in cm from the fixation point that was always present at the center of the screen. Centimeters were chosen because displaying the data in pixels would lead to incomparable results across eye-trackers because the size of pixels of the display screen differed between the systems. To display the data as an angular gaze offset from the fixation point is also not possible because participant distance from the screen varied between eye-trackers and during the instructed head movements and no objective measure of this distance was available.

## Results

### Recovery tasks

The main purpose of the recovery tasks was to determine what happens when an eye-tracker lost track of the eyes and what happens when it starts reporting gaze data again. Some hard to instruct unrestrained participants, such as infants, look away frequently, and it is thus important to know how the eye-tracker deals with this. Before discussing the results of the recovery tasks, we first consider what perfect performance of an eye-tracker on this task would look like. In these tasks, we would expect the reported gaze position to start on the fixation point. After this, we would expect to see the reported gaze position move off the screen as the participants’ started looking away from the screen. Then, we would expect to see the gaze position return toward the screen as the participants rotate their head back to look at the center of the screen. This return to the screen would occur from the same direction in the one direction recovery task, and from the other side of the screen in the 360 recovery task.

**Fig. 2:**
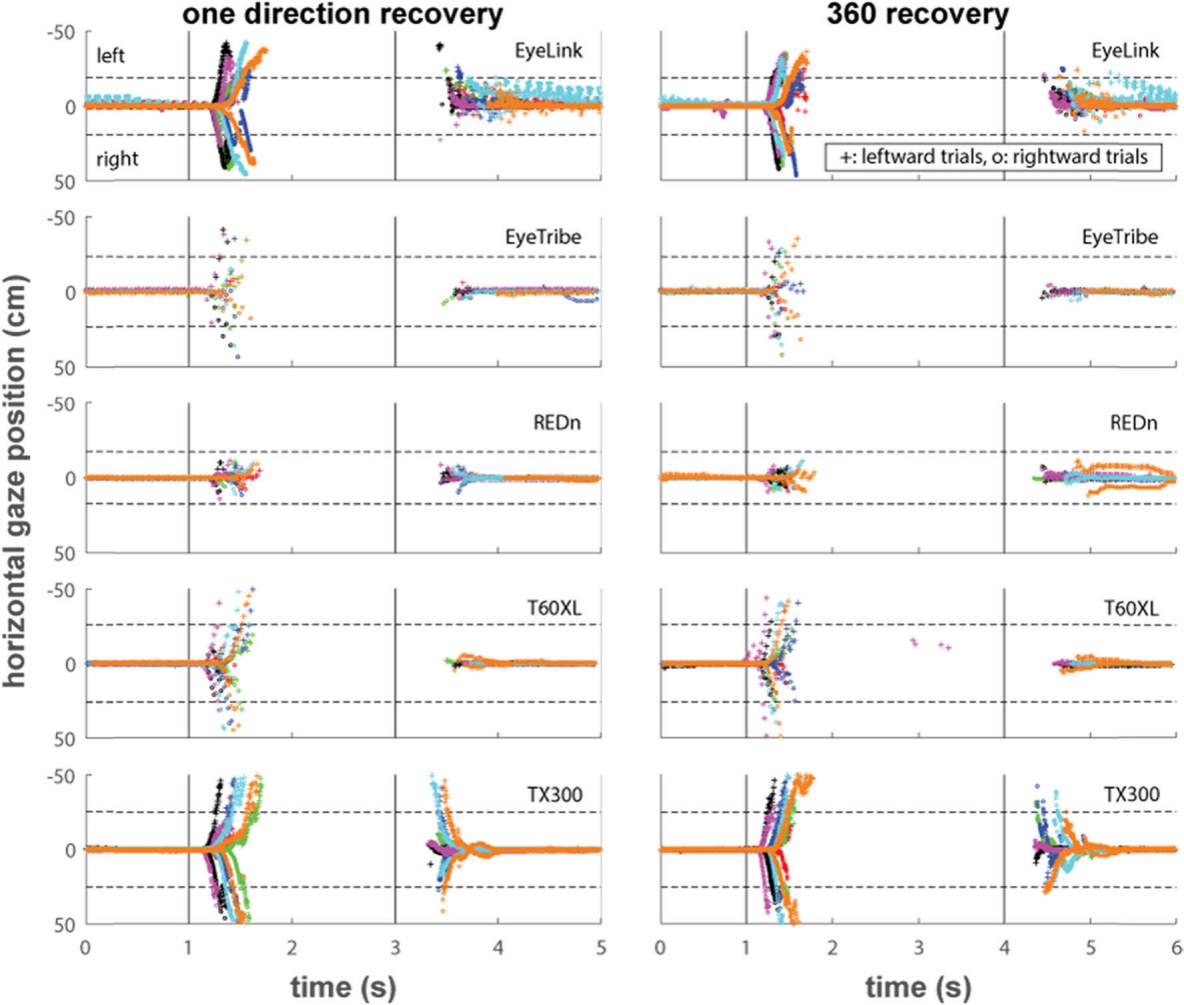
Horizontal gaze position over time for the one direction recovery and 360 recovery tasks. The horizontal dotted lines indicate the edges of the screen, and the solid vertical lines indicate the beeps that instructed the participants to look away from the screen at 1 s and back to screen at either 3 s or 4 s, depending on the task. The plotted data are the average of the raw data of both eyes over ten trials each for looking away to the left (plusses) and to the right (circles)

Figure 2 depicts reported horizontal gaze position for the five eye-trackers in the one direction recovery task (left panels) and 360 recovery task (right panels), averaged for each participant over the ten leftward and rightward trials separately. Only averages for time points at which data for four or more trials was available for a participant are shown. The vertical lines indicate the times at which the instruction beeps sounded, and the dotted horizontal lines indicate the borders of the screen. In general, for both tasks and all eye-trackers, we see that the participants remained fixated at the center of the screen for the first second of the trial. Soon after the first beep sounded, the recorded gaze point moved off the screen. Soon after the second beep sounded gaze data was again recorded at the center of the screen.

Several observations follow from this data. Firstly, all eye-trackers reported gaze coordinates leaving the screen for most or all participants, although it becomes increasingly hard to see for the lower sampling frequency eye-trackers that collected only a few samples during the participants’ head rotation. The SMI REDn frequently lost track before the participants’ gaze reached the border of the screen. Secondly, only the Tobii TX300 managed to reliably pick up the gaze of the participants immediately upon return to the screen, and in some cases before gaze reached the edge of the screen. For some participants, the EyeLink appeared able to track participants’ gaze before it reached the center of the screen, but only when gaze returned from the left side. This is possibly because the camera in the EyeLink setup is positioned such that it has a view from the left side of the eyes instead of perpendicular to the participant. As discussed by Hessels et al. (2015b), an eye-tracker that reports gaze leaving and coming back enables distinguishing between data loss due to a participant looking away from the screen and data loss due to technical difficulties. Knowing when a participant is not looking at the screen can be useful, for instance, when using movies as stimuli that should only play when the screen is attended—these movies can then be paused while the participant looks away. Thirdly, remarkable differences are visible after gaze has returned to the center of the screen. While most eye-trackers delivered stable data indicating fixation at the center of the screen during the last second of the trial, for some of the participants, the EyeLink produced a gaze position signal that jumped between two positions. Further examination revealed that these artifacts even continued into the next trial about 20% of the time. Finally, no obvious differences in performance are seen for any of the eye-trackers between the one direction recovery and 360 recovery tasks.

### Eye-patch task

**Fig. 3:**
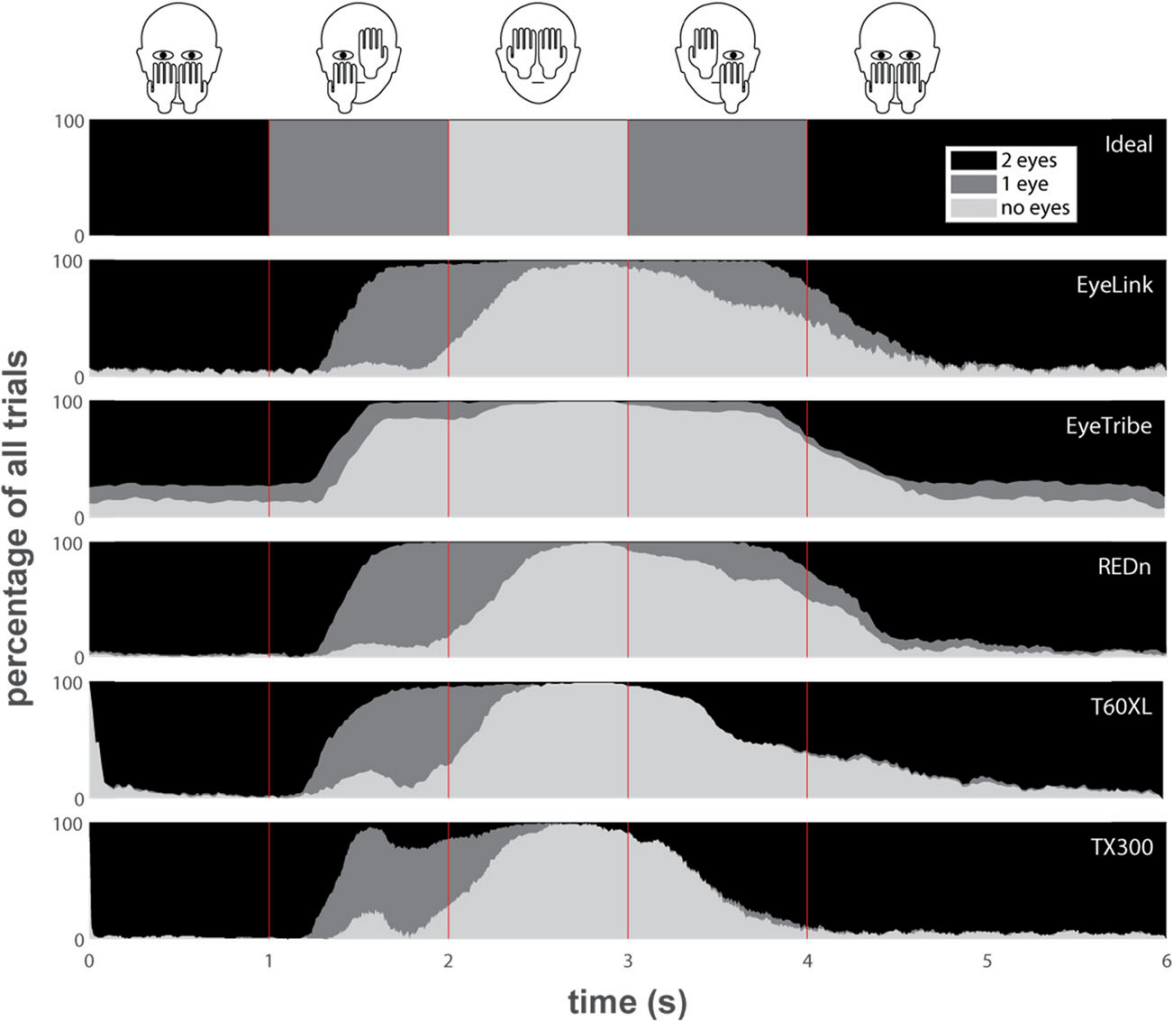
The percentage of all trials from all participants for which the eye-tracker reported data for two eyes (black), one eye (dark grey), or no data (light gray) is plotted over time for each eye-tracker, along with the ideal plot if the eye-tracker performed perfectly and the participants had no delays in their reactions. The vertical red lines indicate the beeps that instructed participants to put up their first hand covering one eye at 1 s, their second hand covering the other eye at 2 s, to lower their first hand uncovering the first-covered eye at 3 s and their other hand uncovering the second eye at 4 s. The schematic above the plots indicates the hand positions during each interval

The main purpose of the eye-patch task was to investigate what happens when the eye-trackers lose track of one eye and later regain tracking of the other eye. We first consider what ideal performance of an eye-tracker in this task would look like (see top panel of Fig. 3). At the start of the task, the eye-tracker should report data for both eyes. Then, as the participants cover their first eye with their hand, the eye-tracker should report gaze data for the remaining eye. When both eyes are covered, the eye-tracker should not report data. Then, when participants uncover their first eye by lowering their hand, the eye-tracker should report data from the eye that reappeared. Finally, the eye-tracker should resume reporting gaze data from both eyes when participants lower their other hand. It should be noted that due to response delay and random variability in the timing of movements by the participants, the data will look shifted in time and smoothed out over time compared to ideal performance.

Figure 3 depicts the percentage of trials of all participants over time for which the eye-trackers reported data for two, one, and zero eyes. Several phenomena are revealed by the data from this task. Firstly, when the first eye is covered for between 1 s and 2 s, most eye-trackers start reporting data for one eye. Only the EyeTribe loses data from both eyes even though only one eye was covered. Secondly, when both eyes are covered for between 2 s and 3 s, all eye-trackers expectedly no longer report data. Lastly, the data during the interval after 3 s reveals two further remarkable phenomena. Firstly, after 3 s when the first eye is uncovered, the EyeLink, EyeTribe, and REDn and to a lesser extent the T60XL continue reporting no data for a majority of the trials, indicating they have difficulty recovering tracking in this case. Secondly, after 3 s, the T60XL and especially the TX300 with very few exceptions never report data from a single eye. Instead they report data from both eyes despite the fact that one eye is still covered by the participants’ hands. For the TX300, a similar phenomenon is seen between 1.5 s and 2 s, where after initially losing track of both eyes as one is covered by the participants’ hands, it resumes reporting data from two eyes for some of the trials.

**Fig. 4:**
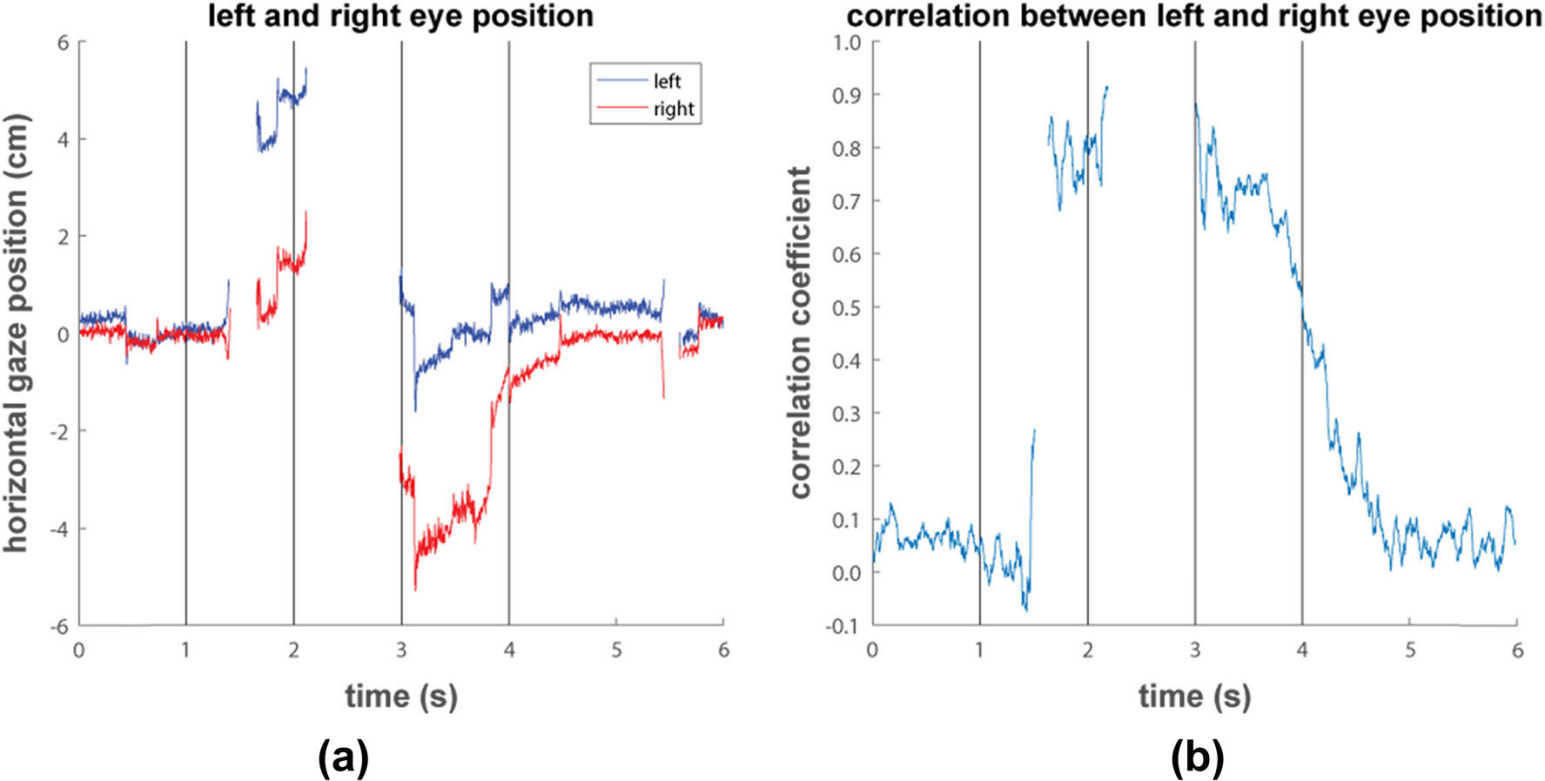
(**a**) Horizontal gaze position during an example trial of the patch task recorded from participant B with the TX300. (**b**) Correlation between the left and the right eye’s horizontal position signal over time as reported by the TX300, averaged over all trials and participants. The vertical black lines indicate the beeps that instructed participants to put up their first hand covering one eye at 1 s, their second hand covering the other eye at 2 s, to drop their first hand uncovering the first-covered eye at 3 s, and their other hand uncovering the second eye at 4 s

To further investigate this appearance of an unexpected second eye, in Fig. 4a we plot the reported horizontal gaze position for the left and right eyes for an example trial recorded with the TX300. The recorded gaze position for both eyes are close to the center of the screen when both eyes are uncovered during the first second of the trial and after 4 s. However, during the intervals between 1.5−2 s and 3−4 s when one of the eyes was covered, the tracker reports data from both eyes with a binocular disparity of 4 cm. It can also be seen that the noise in the position signals from both eyes is more similar during these intervals than before 1 s and after 4 s, which suggests that the data for both eyes originates from the same physical eye. It is possible that the eye-tracker takes the image from the one uncovered eye and processes it separately to produce left and right eye gaze positions from it, where the shared oculomotor and eye-image noise lead to reported position signals that are highly correlated between the two eyes. To see if this occurred systematically when the TX300 reports two eyes while only one eye was visible, we calculated the correlation between the left and right horizontal gaze position signals for each point in time during the trial and plotted this in Fig. 4b. While the gaze position signals were mostly uncorrelated during the first second of the trial and after 4 s when both eyes were visible, correlations were higher for all participants during the intervals between 1.5−2 s and 3−4 s when only one eye was visible. This indicates that it is indeed likely that the TX300 takes the image of a single eye and produces gaze position output for both eyes from it in this test. Similar trends are seen in the vertical position signal and for the T60XL, these are thus not depicted here.

The manual for the Tobii software development kit (Tobii, 2013) gives more insight into what the Tobii eye-trackers do when only one eye is found in the camera image. “*When that happens, the image processing algorithms try to deduce if the eye in question is the left or the right one. This is done by referring to previous eye positions, the position in the camera sensor, and certain image features. […] Validity codes [are provided to] describe the outcome of this deduction.*” Specifically, when one eye is found in the camera image, the validity codes range from 0 to 4 for each eye, with 0 for an eye meaning that the recorded eye is “*most probably*” this eye, 4 meaning it is “*most probably*” not this eye, 2 indicating that the eye-tracker “*cannot with any certainty determine which eye it is,*” and the intermediate codes 1 and 3 indicating intermediate levels of certainty. When one eye is found in the camera image, the validity codes for the two eye channels always sum to 4. This means that if one eye gets a 0, the other is assigned a 4, if one is assigned a 1, the other is assigned a 3, or both eyes can be assigned a validity code of 2. The manual (Tobii, 2013), however, does not clarify what gaze samples the Tobii eye-trackers report when only one eye is found in the camera image. We observe that when the validity codes for the two eyes are 0 and 4, the Tobii eye-trackers only report data for the most likely eye, i.e., the one assigned a validity code of 0. However, when the trackers are not sufficiently certain about which physical eye is recorded from (as indicated by validity codes of 1 and 3 or 2 and 2), we observe that data for both eyes is available in the data file. This may explain why we observe gaze data for two eyes during the interval between 3 and 4 s of the patch task even though we know only one physical eye was visible to the eye-tracker.

**Fig. 5:**
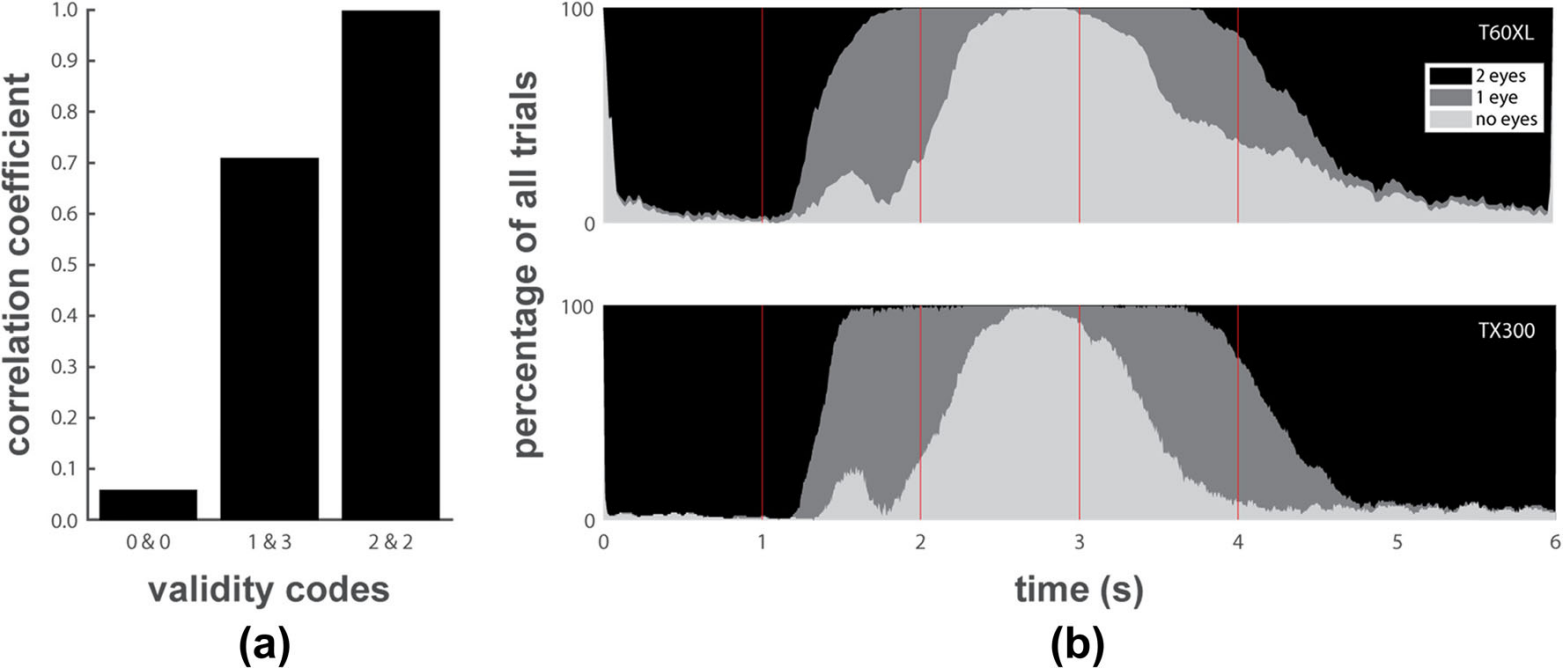
(**a**) The correlation, averaged across participants and time, between the left and the right eye’s horizontal position for data from the Tobii eye-trackers in the patch task when both eyes were in the camera image (as indicated by validity codes of 0 for both eyes), when one eye was found in the image and the eye-tracker is not sufficiently certain which eye it is (validity codes are 1 and 3 for the two eyes), or when one eye was found and the eye-tracker does not know which eye it is (validity codes are 2 for both eyes). (**b**) The proportion of trials for which the eye-tracker reported data for two eyes (black), one eye (dark grey), or no data (light gray) is plotted over time for each eye-tracker for the patch task. The vertical red lines indicate the beeps that instructed participants to put up their first hand covering one eye at 1 s, their second hand covering the other eye at 2 s, to lower their first hand uncovering the first-covered eye at 3 s, and their other hand uncovering the second eye at 4 s

To test whether the data for both eyes reported by the eye-tracker during the patch task when validity codes are larger than 0 actually originated from the same physical eye, we again computed the correlation between the horizontal position signals of the two eyes. We did this separately for when the eye-tracker found two eyes in its camera image (validity codes for both eyes are 0), for when the eye-tracker found one eye and is not sufficiently certain which eye it is (as indicated by validity codes 1 and 3 for the two eyes), and when one eye is found in the camera image and the eye-tracker has no idea which eye it is (validity codes are 2 for both eyes). Inspection of the average correlations across participants and time (Fig. 5a) reveals that correlations are high only when the validity codes are larger than 0, indicating that in these cases the data for both eyes most likely originate from the same physical eye. We thus followed Tobii’s (2013) recommendations to only use data samples with validity codes 0 or 1, i.e., those samples for which the tracker is reasonably certain they are assigned to the correct eye. As such, we coded all samples with validity codes higher than 1 as missing, meaning that when the eye-tracker found a single eye in its camera image as indicated by the validity codes, we only used data for the eye that was designated as most likely by the eye-tracker. Using this selection of data, we replotted the proportion of time over which the eye-tracker reports two, one or zero eyes during the patch task for the Tobii eye-trackers in Fig. 5b. The data now look similar to those from the other eye-trackers and no more data from two eyes is reported when only one eye was visible to the eye-tracker. The only difference from the other eye-trackers is that the T60XL and especially the TX300 frequently manage to report data from one eye after 3 s when the first eye is uncovered, whereas the other eye-trackers only manage to do so for a small subset of trials. As we knew which eye was visible to the eye-tracker during this interval, we were able to establish that the Tobiis correctly designated this eye as the one the eye-trackers were probably recording from. The bump between 1 and 2 s in both panels of Fig. 5b is because both Tobiis temporarily lost track of both eyes when participants B and C raised their first hand.

### Head orientation tasks

The main purpose of the head orientation tasks was to determine whether eye-trackers are able to report gaze data when participants assume non-optimal head poses and, if so, whether systematic offsets occur when the head pose is non-optimal. We first consider what perfect performance of an eye-tracker in these tasks would look like. There should not be any data loss throughout the trial. At the start of each trial, the reported gaze position should be on the fixation point. Then, after the orientation of the participants’ heads was changed, the reported gaze position should remain on the fixation point as the participants continue looking at the fixation point. Finally, after the head has returned to the starting pose, the reported gaze position should still remain at the fixation point. Small changes of reported gaze position may occur during the head movement as the eyes have to correct for the change in head position. As we thus cannot establish whether offsets during this period are due to the participant’s eye movements, or the result of errors in gaze estimation made by the eye-tracker, we will not further discuss offsets that occur during the movement of the head.

**Fig. 6:**
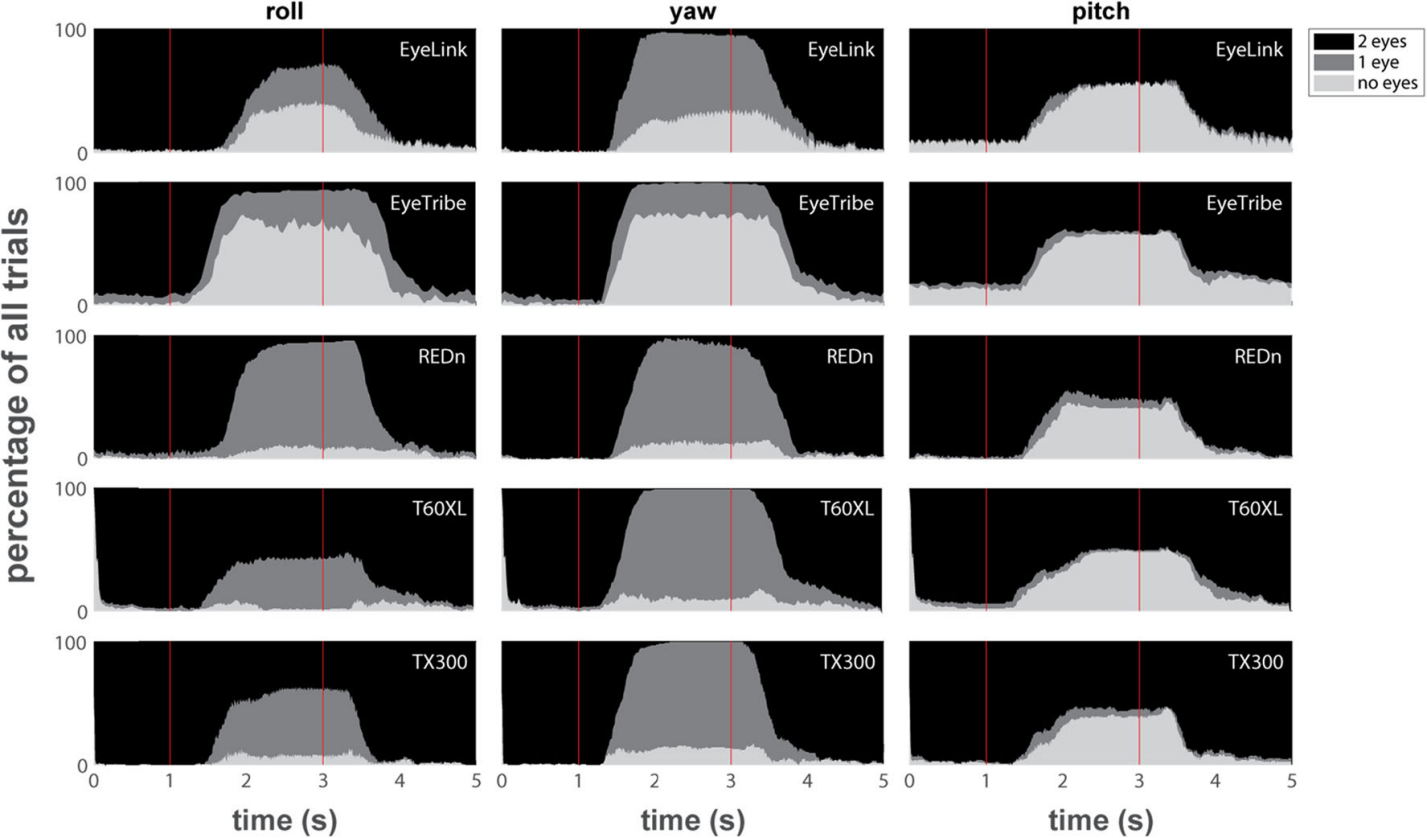
The proportion of trials for which the eye-tracker reported data for two eyes (black), one eye (dark grey), or no data (light gray) is plotted over time for each eye-tracker for the three head orientation tasks. Ideal performance would be to have data from two eyes throughout the trial. The red vertical lines indicate the beeps that instructed the participants to rotate their head while remaining fixated at 1 s and rotate back at 3 s

Figure 6 depicts the number of eyes that the eye-trackers reported data for over time. For the Tobii eye-trackers, samples with validity codes above 1 were coded as missing data (the results of the eye-patch task explain why). For the roll task we see large differences between eye-trackers. While the REDn and the Tobiis are able to report data from at least one eye in most trials, the EyeLink and EyeTribe lose track of both eyes for about half the trials. Similarly, large differences between eye-trackers are seen for the yaw task. Here none of the trackers report data for both eyes when the head is turned away as in this interval one of the eyes is (partially) hidden behind the nasion. However, the trackers differ in whether they are able to report data for one eye. Both Tobii’s and the REDn are able to do so for most trials, while the EyeLink did not report data for close to half of the trials, and the EyeTribe lost track of both eyes in the majority of trials. The data are more consistent across eye-trackers for the pitch task. Here, all trackers report data from two eyes in about half the trials during the interval when the head is turned away. For almost all the other trials, all trackers lost track of both eyes. Further inspection revealed that for all eye-trackers this loss predominantly occurred during trials where the participants titled their head up.

**Fig. 7:**
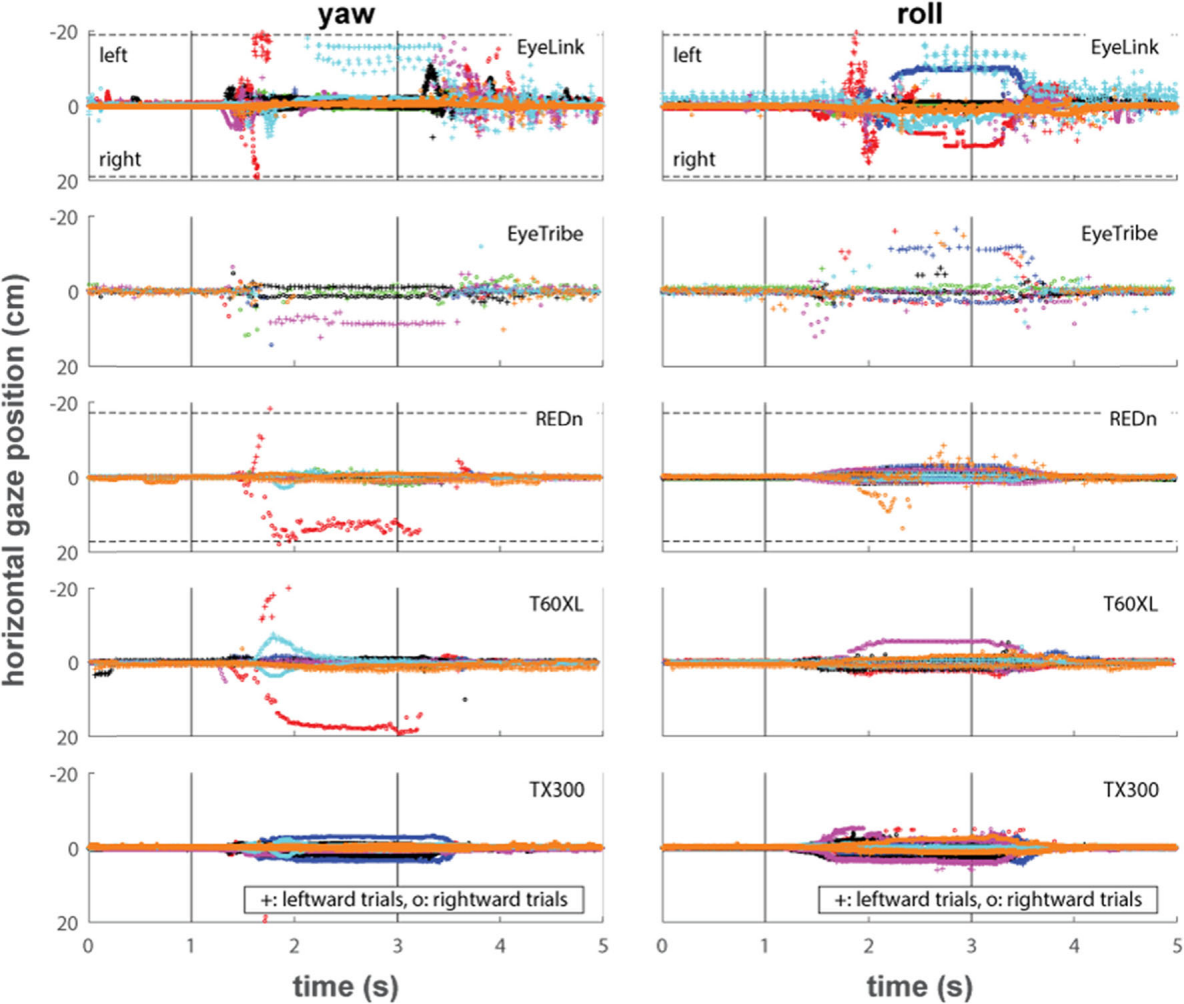
Horizontal gaze positions over time for the yaw and roll tasks. The horizontal dotted lines indicate the edges of the screen (some are outside the plot limits), and the solid vertical lines indicate the beeps that instructed the participants to rotate their head while remaining fixated at 1 s and rotate back at 3 s. The plotted data are the average of the raw data of both eyes over ten trials each for rotating the head to the left (plusses) and to the right (circles)

Figure 7 depicts reported horizontal position data for the yaw and roll tasks when the eye-trackers were able to track the eyes, averaged for each participant over the ten leftward and the ten rightward trials separately. Horizontal gaze position is not shown for the pitch task as head motion in this task did not have a horizontal component. The vertical lines indicate the times at which the instruction beeps sounded telling the participants to rotate their head, and the dotted horizontal lines indicate the borders of the screen. Although data for the EyeLink contain multiple artifacts, no more than small offsets are seen in all trackers for most participants when the head is rotated during the yaw task. Offsets in horizontal gaze position are more pronounced during the roll task, and are especially large for some participants on the EyeLink and the EyeTribe.

**Fig. 8:**
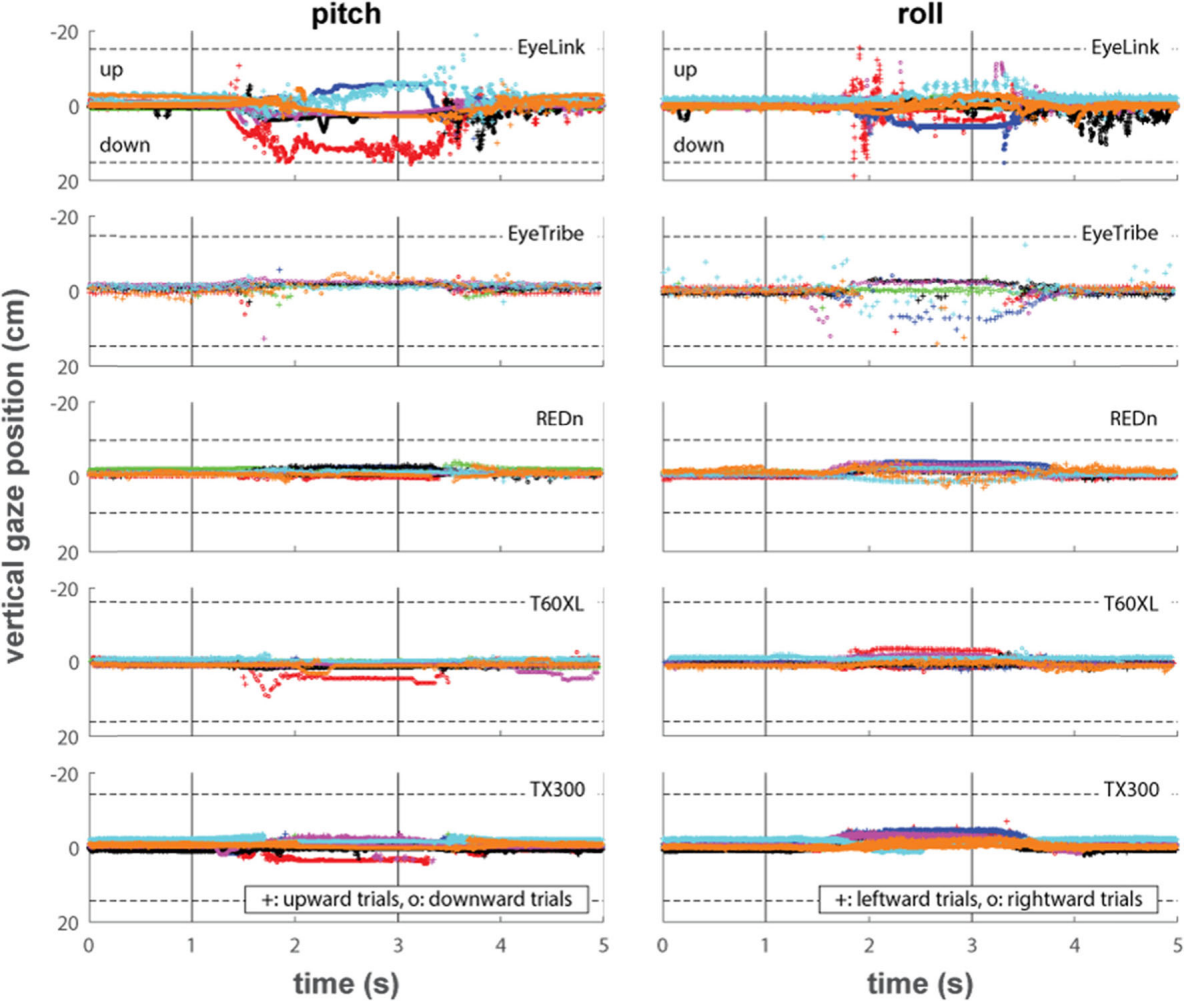
Vertical gaze positions over time for the pitch and roll tasks. The horizontal dotted lines indicate the edges of the screen, and the solid vertical lines indicate the beeps that instructed the participants to rotate their head while remaining fixated at 1 s and rotate back at 3 s. The plotted data are the average of the raw data of both eyes over ten trials each for rotating the head to the left or up (plusses) and to the right or down (circles)

Figure 8 depicts reported vertical position data for the pitch and roll tasks when the eye-trackers were able to track the eyes, averaged for each participant over the ten left- or upward and the ten rightward trials separately. Vertical gaze position is not shown for the yaw task as head motion in this task did not have a vertical component. During the pitch task, most eye-trackers report data with only small vertical gaze position offsets for most participants. Only the EyeLink shows large systematic vertical gaze position offsets for most of the participants. The pattern of data for vertical gaze position during the roll task is similar to that for horizontal gaze position; all eye-trackers produce offsets during this task, with the EyeLink and EyeTribe producing especially large offsets for some of the participants. It can furthermore be seen from all panels in Figs. 7 and 8 that the EyeLink produces large short-lived artifacts around the time of head rotation.

## Discussion

Eye-tracker manufacturers suggest in their marketing materials that as long as a participant is inside the eye-tracker’s headbox, the remote eye-tracker will be able to track the participant’s gaze and deliver high quality data. However, our experience with infants, who look away from the screen frequently and who cannot be restrained allowing them to take on non-optimal head poses, is that data quality can vary strongly depending on the participant’s orientation. This is the case even when the infants’ eyes remain in the eye-tracker’s headbox and they are looking at the screen. This article was inspired by this discrepancy between the manufacturers’ marketing materials and what we observe in practice. A selection of popular and current eye-trackers was put through a series of tests that modeled behaviors that we can expect from unrestrained participants. Specifically, in our tests the participants looked away from the screen and back, covered one or two eyes, and rotated their heads along one of three axes. Understanding how the eye-trackers in our test perform will help researchers working with unrestrained participants understand what to expect from their eye-tracker in these circumstances, and help them make an informed decision about what eye-tracker is suitable for their experiment. Our data furthermore help researchers understand the constraints posed on the design of their experiment by the data quality of the eye-tracker during non-optimal head positions. As such, our results are relevant for work in any field where recordings are made from participants that cannot be restrained or instructed to sit in the optimal pose during eye-tracking, such as for instance researchers working with infants, school children, ADHD patients, or clinical groups such as Alzheimer and Parkinson patients and people with muscular disorders. Below, we will further discuss each of the five eye-trackers that took part in our study.

The EyeLink 1000Plus was included in our tests as it is increasingly used as a remote eye-tracker and because it is advertised as providing the highest spatial precision and accuracy among video-based eye-trackers. Our tests, however, show that the EyeLink in its remote eye-tracker setup has large problems when participants took on non-optimal poses. Specifically, the EyeLink produced large horizontal and vertical gaze offsets in the head-orientation tasks. Offsets in eye-tracking data can invalidate a study’s findings. Holmqvist et al. (2012) have previously shown that offsets in the reported gaze coordinates as small as 0.5° can strongly alter a study’s results. Although recent work (Hessels et al., 2015c) has shown that the effect of spatial offsets in eye-tracking data on the outcome measures of a study depends on how AOIs are constructed, the accuracy of an eye-tracker and the offsets it may produce given the mobility of the population of interest should be taken into account when designing stimuli (see Holmqvist et al., 2011 for a detailed discussion). It should further be noted that the EyeLink was the only tracker in our test that used a sticker on the participant’s forehead to help in determining how the participant’s head is positioned—all other eye-trackers used solutions that did not require the addition of marker to the participant. Although this sticker may help the EyeLink to compensate for head motion and to locate the eyes in the camera image, we have also observed it to lead to problems in some cases. For one participant with longer hair, we had to be careful before the start of each task that his hair did not occlude the sticker. The EyeLink also appears to use the spatial relation between the sticker and an eye in the camera image to determine which eye it is recording from. During the roll task especially, this led to problems. As the head made a roll rotation in the camera image, the spatial relation between the sticker and the location of the eyes in the camera sometimes changed to such an extent that the eye-tracker was only able to record from one eye, and incorrectly determined which eye this was. Last, it should be noted that when using the EyeLink in the setup recommended in the manual, we found it to have problems tracking the eyes when participants looked at the calibration points on the left side of the screen. This could have been because the screen was too large despite following the recommendations in the manual for screen size and viewing distance, or because the infrared illuminator in this setup was too far to the right which we observed to sometimes lead to problems with identifying the corneal reflection (due to smearing as it left the corneal bulge). We additionally recorded data for all six tasks from three of the participants with a setup altered such that the infrared illuminator was placed less far to the right of the participants and with the participants at 70 cm instead of 65 cm from the eye-tracker. The data from the three participants in the altered setup, however, reproduced all the major findings reported above.

The EyeTribe also suffered significant problems in our test. As soon as participants’ heads are in non-optimal orientations, it suffers from significant data loss. Furthermore, we found that the sampling rate at which it reported data was not stable. The rate at which it reports gaze data appears to alternate between intersample intervals of approximately 33 ms and 44 ms, corresponding to sampling rates of approximately 30 Hz and 23 Hz. A non-constant intersample interval can lead to difficulties in determining eye-velocities, which are used by some fixation detection algorithms (e.g., Hooge & Camps, 2013) to determine when and for how long the eye rests at a certain position on the screen (see Hessels et al., 2015b for further discussion). A failure to take non-constant intersample intervals into account can furthermore invalidate all measures based on the timing or duration of eye-movement events, such as the latency until participants first look at an area of interest and the total time spent looking at it.

Except for its inability to report data when gaze left and reentered the screen, the REDn did not suffer from major problems in our tests. Hessels et al. (2015b) previously reported that the RED-m, the REDn’s predecessor, and another SMI eye-tracker in their test, the RED250, suffered from three significant problems. Firstly, it was found that the RED-m sometimes confused the two eyes (i.e., e.g., the left eye was processed as if it were the right eye), leading to systematic large offsets in gaze position during the yaw task. Secondly, it was found that the RED-m and the RED250 suffered from drops in sampling frequency down to 20 Hz which started approximately half a second after the trackers lost track of the eyes. Lastly, Hessels et al. (2015b) found that the RED250 suffered data loss in about 60% of the trials during the yaw task when the head was rotated away from the screen but the eyes maintained fixation at the center of the screen. Our tests revealed that the REDn produces low data loss in the yaw task, and we found no evidence of a drop in sampling rate when the eye-tracker was unable to find the eyes in its camera image. However, we did find some evidence for eye confusion in the gaze position signals during the 360 recovery and patch tasks, as was for instance visible in our data as the consistent large offset in reported gaze position for some of the participants in the 360 recovery task (see Fig. 2). Specifically, although it only occurred for two participants, we saw large and persistent gaze offsets after 3 s when the first eye was uncovered in the patch task, and after 4 s when participant’s gaze returned back to the screen in the 360 recovery task. These offsets are likely due to eye confusion in the eye-tracker. The REDn was the only eye-tracker in our test that in its default setup is attached to the bottom of a laptop screen. As such, it was positioned much lower with respect to the participant’s head and eyes than the other 4 eye-trackers, which filmed the eyes from a vertical position that was much closer to the eyes. In a further test, we therefore attached the REDn under a desktop flatscreen and recorded data for all six tasks from three of the participants. The results were largely the same as reported above, except that the spread in horizontal and vertical gaze coordinates across trials and participants decreased by about half.

The T60XL was included in our test because its manufacturer, Tobii, said it was their best machine for tracking mobile participants as it has the largest headbox among its products. However, in this study we found the T60XL to perform very similar to the TX300 on all tests, with two exceptions. First, horizontal position offsets during the yaw and roll tasks were smaller overall on the T60XL than on the TX300. Second, the T60XL did not show gaze returning to the screen in the recovery tasks, whereas the TX300 did. Given the small differences between the T60XL and TX300, the TX300 is perhaps the more flexible choice as its higher precision and higher sampling frequency enables detecting eye-movement events more precisely, and decreases how much data are lost when one or two sample bursts of data loss occur, such as are seen in recordings performed with infants (Hessels et al. 2015a). The TX300 performed similarly well in our tests as it did in Hessels et al. (2015b). It showed small offsets during the head orientation tasks, and robust recovery when eyes reappeared in the camera image as evidenced by the gaze returning back to the screen in the recovery tasks and having data for one eye for a majority of trials in the patch task when the first eye was uncovered after 3 s.

Our tests have revealed that in certain circumstances, the Tobii eye-trackers report data for both eyes even though the eye-tracker found only a single eye in its camera image. Importantly, the manual does not specify that data for two eyes may be reported. It only indicates that each reported gaze position is supplemented with a so-called validity code, indicating how sure the eye-tracker is about which eye it is recording from. While the manual indicates only using gaze position samples for which the eye-tracker reports it is reasonably certain that it has identified the correct eye, researchers who expect bad data quality, for instance because of highly mobile participants, may well decide to use all data the eye-tracker is able to provide. Our tests indicate that choosing to do so may lead these researchers to use data where the eye-tracker reports a gaze position for two eyes even though only one eye was visible to the eye-tracker. In our tests, this situation occurred between 2% (for the recovery tasks and the pitch task) to 9% (yaw task) and even 14% (roll and patch tasks) of all the gaze samples recorded with the TX300 (for the T60XL, this ranged between 1% and 8%). Furthermore, large consistent offsets are present in the gaze position reported by the eye-tracker when the eye-tracker reports data from one eye as if it were two eyes (see Fig. 4a). As such, we think that Tobii has made a dangerous decision in providing *by default* these probabilistic gaze samples where the researcher him- or herself has to choose which eye the data came from. We question whether researchers are able to make this decision better than the eye-tracker, given that they generally have no information other than the validity code provided by the eye-tracker to decide which eye was recorded. Providing these probabilistic gaze samples could be an interesting feature to be enabled in special situations where researchers are able to acquire extra information about which eye was visible to the eye-tracker. However, in its current setting and combined with the lack of documentation that data for two eyes is reported, it is likely to lead researchers to use incorrect gaze samples with large offsets in reported gaze position, which will decrease a study’s power to find results, or in the worst case, even invalidate a study’s results. Given that we have seen such data reported for up to 14% of all samples in some of our tasks, we furthermore urge researchers currently using the Tobii eye-tracker to be careful in selecting data for further analysis.

In conclusion, the results of our tests have shown that eye-trackers may have significant trouble tracking participants in non-optimal conditions, even though the participants’ eyes remain in the headbox. This finding underscores the importance of not only looking at the manufacturers’ specifications when deciding which eye-tracker to buy for your experiment, but to also consider and, when possible, test the eye-tracker in the conditions in which your experiment will likely take place. Both the previous study that tested remote eye-tracker performance in non-optimal conditions (Hessels et al., 2015b) and this study have furthermore uncovered problems in the eye-trackers that easily lead to systematical errors in data analysis if the researcher is not aware of them. As such, the series of qualitative tests we proposed in this article are not only useful for researchers to evaluate their equipment, but also for manufacturers when testing their prototypes. We hope that manufacturers will use our tests during the development of new remote eye-trackers to make their machines more robust to non-optimal conditions, and able to live up to their promise of position and orientation-invariant data quality as long as the participant’s eyes are within the eye-tracker’s headbox.
